# Evaluating tactile feedback in addition to kinesthetic feedback for haptic shape rendering: a pilot study

**DOI:** 10.3389/frobt.2024.1298537

**Published:** 2024-04-10

**Authors:** Alexandre L. Ratschat, Bob M. van Rooij, Johannes Luijten, Laura Marchal-Crespo

**Affiliations:** ^1^ Motor Learning and Neurorehabilitation Lab, Department of Cognitive Robotics, Delft University of Technology, Delft, Netherlands; ^2^ Department of Rehabilitation Medicine, Erasmus MC, University Medical Center Rotterdam, Rotterdam, Netherlands; ^3^ SenseGlove, Delft, Netherlands

**Keywords:** VR training, haptic display, haptic rendering, kinesthetic devices, tactile devices, virtual reality

## Abstract

In current virtual reality settings for motor skill training, only visual information is usually provided regarding the virtual objects the trainee interacts with. However, information gathered through cutaneous (tactile feedback) and muscle mechanoreceptors (kinesthetic feedback) regarding, e.g., object shape, is crucial to successfully interact with those objects. To provide this essential information, previous haptic interfaces have targeted to render either tactile or kinesthetic feedback while the effectiveness of multimodal tactile and kinesthetic feedback on the perception of the characteristics of virtual objects still remains largely unexplored. Here, we present the results from an experiment we conducted with sixteen participants to evaluate the effectiveness of multimodal tactile and kinesthetic feedback on shape perception. Using a within-subject design, participants were asked to reproduce virtual shapes after exploring them without visual feedback and with either congruent tactile and kinesthetic feedback or with only kinesthetic feedback. Tactile feedback was provided with a cable-driven platform mounted on the fingertip, while kinesthetic feedback was provided using a haptic glove. To measure the participants’ ability to perceive and reproduce the rendered shapes, we measured the time participants spent exploring and reproducing the shapes and the error between the rendered and reproduced shapes after exploration. Furthermore, we assessed the participants’ workload and motivation using well-established questionnaires. We found that concurrent tactile and kinesthetic feedback during shape exploration resulted in lower reproduction errors and longer reproduction times. The longer reproduction times for the combined condition may indicate that participants could learn the shapes better and, thus, were more careful when reproducing them. We did not find differences between conditions in the time spent exploring the shapes or the participants’ workload and motivation. The lack of differences in workload between conditions could be attributed to the reported minimal-to-intermediate workload levels, suggesting that there was little room to further reduce the workload. Our work highlights the potential advantages of multimodal congruent tactile and kinesthetic feedback when interacting with tangible virtual objects with applications in virtual simulators for hands-on training applications.

## 1 Introduction

Virtual reality (VR) based training is commonly used in, e.g., medical (van der Meijden and Schijven, 2009) and industrial education (Carlson et al., 2015) due to its cost-effectiveness, ethical considerations, and safety. VR provides an excellent environment to practice and learn new skills since, in the early stages of learning, basic movements and movement sequences are learned using visual feedback following a steep learning curve (van der Meijden and Schijven, 2009). Yet, the skillful interaction of humans with real-world objects requires not only visual feedback but also information gathered via the mechanoreceptors in our skin (tactile feedback) and muscles (kinesthetic feedback), collectively termed as haptic feedback.

Haptic feedback has been shown to improve fine motor control (Danion et al., 2012) and is associated with better motor learning when compared to visual feedback alone (Özen et al., 2022). Adding haptic feedback during VR-based training might also increase the realism of the virtual task at hand, enhancing the user’s motivation and favoring the transfer of skills gained in virtual environments (VEs) to real life (IJsselsteijn et al., 2006; Levac et al., 2019). To leverage these benefits, advancements have been made to provide haptic feedback during VR-based training—an overview of research and commercial haptic displays can be found in the reviews by Pacchierotti et al., and Perret and Vander Poorten (Pacchierotti et al., 2017; Perret and Poorten, 2018). Haptic feedback has been employed in combination with VR, e.g., to improve performance in teleoperation (Nitsch and Farber, 2013), to reduce errors and improve performance in minimally invasive surgery (van der Meijden and Schijven, 2009), and as an aid for micromanipulation and microassembly (Bolopion and Regnier, 2013).

We can find dedicated solutions to provide either tactile or kinesthetic feedback. Commercial solutions to provide tactile feedback include, e.g., the fingertip-mounted device weart touchdiver (Weart S.r.l., Italy), which provides tactile feedback with normal forces to the fingertip, simulating making and breaking contact with a virtual surface, and thermal cues. Furthermore, tactile feedback devices are applied in teleoperated surgery (Pacchierotti et al., 2016), weight rendering of virtual objects (Minamizawa et al., 2007), or softness simulation in augmented reality surgical training (Fani et al., 2018). Examples of commercial devices that provide kinesthetic feedback include tabletop haptic robotic devices like the Delta.3 (Force Dimension, Switzerland) and wearable glove-like solutions, such as the SenseGlove Nova (SenseGlove, Netherlands). These kinds of devices can render kinesthetic feedback on multiple fingers or the whole hand for applications ranging from virtual assembly tasks (Zheng et al., 2021) to surgical training (Gani et al., 2022) and 3D modeling and design (Liu et al., 2004). Yet, only a few solutions, such as the HaptX Gloves G1 (Haptx Inc., United States), are capable of providing both tactile and kinesthetic feedback despite the potential benefit of combining both types of haptic feedback to enhance VR-based skill training.

In addition to the above-mentioned examples using haptic feedback to render virtual object characteristics—i.e., weight, deformations, surface texture, stiffness, and shape—we can find extensive literature supporting the benefit of haptically rendering virtual objects’ characteristics using either tactile or kinesthetic feedback on the perception and manipulation of virtual objects. For example, Leonardis et al. found significantly reduced maximum peak and interaction forces in a lift-and-hold task when applying tactile feedback with visual feedback compared to visual feedback alone (Leonardis et al., 2017). Chinello et al. found that the users’ stiffness discrimination enhanced when using tactile feedback in combination with vibrotactile feedback, compared to vibrotactile feedback alone (Chinello et al., 2018). However, we found less literature regarding the perception of virtual objects’ shapes, an important characteristic to perceive when identifying and manipulating objects. Perhaps the most remarkable study is the one from Dostmohamed and Hayward, who showed that it was possible to discriminate curvatures with a two-degree-of-freedom table-top servo-controlled tactile display (Dostmohamed and Hayward, 2005).

Notably, the potential benefit of congruently combining tactile and kinesthetic feedback in shape perception has been rarely explored. Addressing this gap, Frisoli et al., 2011 investigated the angle perception between two surfaces with either tactile or kinesthetic feedback and with the combination of both. They found that multimodal haptic feedback led to better discrimination performance than each single feedback alone. In a recent study, Suga et al., 2023 compared 3D edge detection with electro-tactile stimulation, kinesthetic feedback, and a combination of both. The kinesthetic and combined feedback conditions consistently outperformed the electro-tactile feedback condition, and the combined feedback condition showed the shortest identification times. These results support the idea that multimodal haptic feedback might enhance the perception of discrete surface edges. Yet, it is still an open question whether multimodal haptic feedback could also enhance the perception of more complex continuous shapes—such as those of virtual objects with complex geometrical shapes—and whether users could use this more enriched information to reproduce the explored shapes without any feedback.

In this work, we investigated the performance of sixteen healthy participants in reproducing virtual shapes after exploring them with kinesthetic feedback vs. kinesthetic with additional tactile feedback, both without visual feedback. An adapted SenseGlove Nova mechanism was utilized to provide the kinesthetic feedback, while the tactile feedback was rendered using a spring-loaded two-degree-of-freedom (DoF) platform at the fingertip actuated via two wires independently and remotely tensioned by two motors. By independently controlling the tension of the wires, the platform could be rotated with respect to the longitudinal axis of the finger, allowing the rendering of different virtual shapes, the interaction forces, and the making and breaking of contact. To evaluate whether training with the different haptic conditions affected the participant’s workload and motivation, these physiological effects were evaluated using standardized questionnaires. While a high mental workload may overwhelm the participants and thus hamper their learning capacities (Meyer et al., 2019), enhanced motivation can facilitate motor learning (Wulf and Lewthwaite, 2016).

We hypothesized that participants would reproduce the virtual shapes more accurately and spend less time exploring those during training when provided with multimodal tactile and kinesthetic feedback compared to kinesthetic feedback alone. Additionally, we hypothesized that participants would report higher motivation levels and less workload when practicing with the additional tactile feedback, mainly driven by the more informative feedback and perceived competence.

## 2 Methods

### 2.1 Participants

Sixteen healthy participants provided written informed consent to participate in the study (eight female and eight male, all right-handed, 21–35 years old, mean age 26.3). All participants were naive to the haptic device used in the experiment. The study was approved by the TU Delft Human Research Ethics Committee (HREC, Application ID 2199) and was conducted in compliance with the Declaration of Helsinki in SenseGlove, Netherlands.

### 2.2 Shape exploration experiment

**FIGURE 1:**
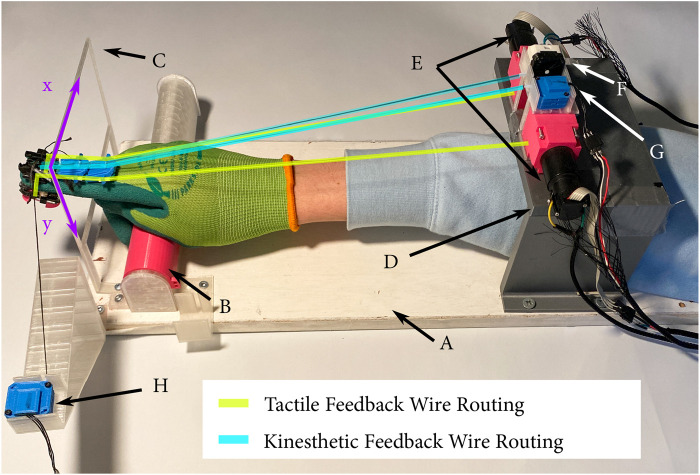
The test bench setup for the validation experiment including a participant’s arm, showing the **(A)** base plate, on which the participant’s arm is placed; **(B)** slider where the participant could rest the palm; **(C)** frame representing the 2D-workspace for the experiment with the coordinate frame on the left top corner; **(D)** mounting platform for the actuation module, containing the **(E)** motors, the **(F)** electromagnetic brake, and the **(G)** hall-effect sensor measuring the *y*-position for the fingertip; and **(H)** mount for the hall-effect sensor measuring the x-position of the fingertip.

We investigated whether participants could reproduce different two-dimensional shapes in free space after exploring the shapes with two haptic feedback conditions: 1) kinesthetic feedback only (K) and 2) kinesthetic plus tactile feedback (KT). The participants explored the shapes with their right index fingertips by approaching the shapes from the top within a two-dimensional rectangle with an area of 135 × 80 mm (Figure 1). A custom device added to a modified Nova glove (SenseGlove, Netherlands; see Section 2.4.2) provided tactile feedback regarding shape contour and surface contact. The modified Nova mechanism delivered the kinesthetic feedback by braking the vertical finger movement when the finger touched a shape from the top (see Section 2.4.3).

Each participant tested the two conditions (within-subject design) in a randomized and counterbalanced order with one experimenter present at all times. Before beginning the experiment, the participants received written instructions on the three phases of the experiment for each condition: The familiarization phase, the experimental test phase, and the evaluation phase. Furthermore, the participants were asked to don the modified Nova mechanism and received instructions on how to perform the exploration and reproduction, i.e., by approaching shapes from the top while exploring and reproducing shapes in one smooth motion from left to right. Participants were neither blindfolded nor wore headphones during any part of the experiment.

**FIGURE 2:**
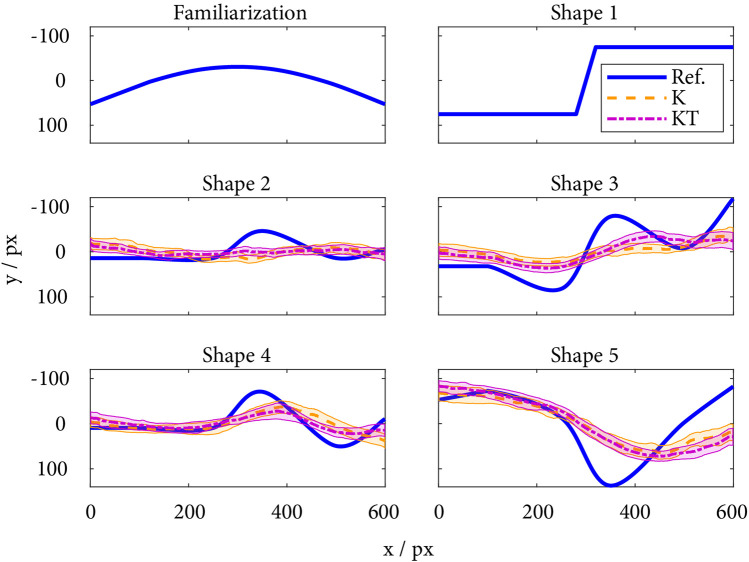
The reference shapes rendered during the familiarization phase and the exploration trials (Ref.), and the mean and CIs of the reproductions per condition (K and KT) for shapes 2–4. The mean was subtracted for both the reference and reproduced shapes. Note that the exploration and reproduction of Shape 1 were also considered familiarization. The *x* and *y*-axes correspond to the rectangular area of the experimental setup, which is divided into pixels (px) corresponding to the shape representation on the screen (only shown during the first familiarization phase).

For each condition, there was a 120 s familiarization phase using the familiarization shape (Figure 2). Here, the participants could freely explore the shape with the condition’s respective haptic feedback while visualizing the shape and the position of the fingertip on a computer screen. After the familiarization phase, we turned off the shape visualization, and participants started with the experimental test phase.

During the experimental test phase, the participants explored and reproduced five different shapes (Figure 2, Shapes 1–5) without visual feedback on the computer screen—they could, however, see their finger within the rectangular area of the experimental setup. For each shape, participants were asked to first explore the shape with the respective kind of feedback at their own pace without a time limit and to communicate with the experimenter once they felt ready to reproduce the explored shape. They were then asked to reproduce the perceived shape by moving their index finger from left to right within the rectangular workspace while the feedback was deactivated. The exploration and reproduction were repeated three times (trials) per shape, leading to 15 reproduction measurements per condition (3 trials x 5 shapes). The presentation order of the shapes was kept the same for all participants and conditions (Shape 1 first—Shape 5 last). We considered the exploration and reproduction of the first shape as familiarization, as it was the first time participants had to reproduce a shape without feedback.

Finally, after each condition’s evaluation phase, participants were asked to fill out two questionnaires, i.e., the Raw Task Load Index (RTLX) (Hart, 2006) and the Intrinsic Motivation Inventory (IMI, subcategories: perceived competence, effort/importance, interest/enjoyment) (McAuley et al., 1989).

### 2.3 Shape design

We defined six different shapes with diverse characteristics (Figure 2). A simple concave shape was chosen as the Familiarization Shape to introduce the participants to the experimental procedure. Shape 1 was designed as a simple ramp. The remaining shapes (Shapes 2–5) were defined as a combination of smooth curves with a different number of maxima and minima and different amplitudes and, therefore, varying complexity. Shapes 2–4 were chosen with a similar basic contour in mind, slightly modified for each shape to challenge the participants’ fine haptic perception with the two haptic conditions. All shapes were characterized by six individual points spread along the *x*-axis, where the first and last points were always at *x*_0_ = 0 px and *x*_5_ = 600 px, respectively. The *y* coordinates of the points were chosen between *y*_*i*_ ∈ [40, 340] px. One pixel corresponds to 0.225 mm and 0.2 mm in *x* and *y* of the exploration area, respectively. For Shape 1, straight lines connected the points, and the remaining shapes were interpolated with Catmull-Rom splines.

### 2.4 Experimental setup

#### 2.4.1 Test bench

The experiment was conducted using the test bench illustrated in Figure 1. The test bench included a mounting platform located above the user’s forearm where the actuators for the tactile and kinesthetic feedback and the sensors to record the fingertip position were placed (denoted as *d* in Figure 1). Participants were free to move their index fingertips from left to right within the two-dimensional rectangle that represents the experimental area (denoted as *c* in Figure 1). We included a slider that can move along the long side of the rectangle to provide support to the palm and prevent fatigue while participants move their hands laterally (denoted as *b* in Figure 1).

#### 2.4.2 Mechanical design of the tactile feedback mechanism

**FIGURE 3:**
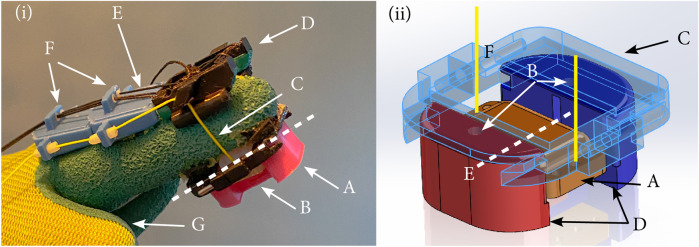
Overview of the tactile feedback fingertip mechanism **(i)**: **(A)** Feedback module mounted on the fingerpad, **(B)** moving platform that contacts the finger pulp, **(C)** wire connecting the platform to the actuator, **(D)** mounting module to guide the actuated wires with also an attachment point for the wires from the altered SenseGlove Nova, **(E)** wires of the SenseGlove Nova, **(F)** guiding modules on the intermediate and proximal phalanges leading the wires toward the actuation module and **(G)** glove used to don the device. Close-up CAD rendering of the tactile feedback module **(ii)**: **(A)** Moving platform with holes to attach the wires, **(B)** holes for the two springs forcing the platform away from the finger, **(C)** bracket attached to the soft glove, **(D)** guides for the platform and springs, **(E)** mobile platform axis of rotation, and **(F)** wire path.

We developed a fingertip-mounted mechanism to provide tactile feedback, which could be used alongside the kinesthetic feedback mechanism of the SenseGlove Nova (see Section 2.4.3). The mechanism had two degrees of freedom for haptic interactions: one rendered the making and breaking of contact with virtual objects, and the other simulated their shapes by rendering the contact angle around the proximodistal axis of the index finger. This was achieved using a small 3D-printed (polylactide, PLA) spring-loaded platform (1.5 × 5.7 mm, with two compression springs of approx. stiffness 400 Nm^−1^, denoted as *b* in Figure 3i) mounted on the ventral side of the finger, which was in direct contact with the skin. A close-up CAD rendering is depicted in Figure 3ii. The platform was actuated via two mechanical wires (spiderwire, diameter 0.33 mm, max. load 38.1 kg, A.C. Kerman, Inc., United States) connected to the ulnar and radial side of the fingertip platform (denoted as *c* in Figure 3i).

We opted for a wire-based actuation mechanism for several reasons, i.e., the availability of the wire infrastructure from the Nova, and the lightweightness and compactness of the mechanism on the fingertip. Two DC motors (2342S048CR, Faulhaber, Germany) with integrated encoders (IE3-32, Faulhaber, Germany) and matching pulleys (diameter 14 mm) on the mounting platform controlled the wires’ tensions. The pulleys were pre-tensioned by a constant-force spring attached to the motor housing to avoid slacking of the wires. The wires were guided from the fingertip platform to the motors using a second 3D-printed module mounted on the dorsal side of the fingertip (denoted as *d* in Figure 3i) and two 3D-printed guiding modules on the intermediate and proximal phalanges attached to a soft glove worn by the user (denoted as *f* in Figure 3i). The area of the soft glove where the platform made contact with the fingerpad was cut off to enhance the tactile sensation.

To simulate making contact with the virtual shape, the DC motors tensioned the cables to move the platform toward the finger. Here, the springs attached to the platform ensured that the contact was broken once the tensions of the wires were released. To render the geometry of the shape, the tensions of each wire were controlled independently to rotate the platform along the longitudinal axis of the finger (denoted as a dashed line in Figure 3ii) and orient the mobile platform (denoted as *a* in Figure 3ii) tangential to the shape at the contact point. The maximum achievable rotation of the fingertip platform was 30° in either direction.

The designed tactile feedback mechanism, i.e., the spring-loaded platform on the ventral side of the finger and guiding module on the dorsal side, had a total weight of 7 g and a size of 30 × 27 × 16 mm (width x length x height), excluding the thickness of the user’s fingertip.

#### 2.4.3 Mechanical design of the kinesthetic feedback mechanism

The kinesthetic feedback mechanism was designed to stop the flexion of the index finger when touching a virtual shape from the top, simulating shape contact. This was realized via an adapted one-degree-of-freedom SenseGlove Nova mechanism with an integrated electromagnetic brake (custom brake ATD7883, max. 5 N brake force, SG Transmission, UK). Two wires (same specifications and pre-tensioning system as for the tactile mechanism) connected the electronic brake on the mounting platform (denoted as *f* in Figure 1) to the dorsal side of the distal phalanx of the index finger via guides on the intermediate and proximal phalanges (Figure 3i).

#### 2.4.4 Fingertip position sensing

The two-dimensional position of the fingertip within the rectangular experimental workspace was measured using two hall-effect sensors (AS5600, ams-OSRAM AG, Austria), each connected to the fingertip with a wire (same specifications and pre-tensioning system as for the tactile mechanism). A first hall sensor, located at the mounting platform next to the DC motors (denoted as *g* in Figure 1), measured the length of the SenseGlove Nova wire used in the kinesthetic feedback mechanism connected to the mounting module. The second hall sensor was mounted on a beam lateral to the experimental area (denoted as *h* in Figure 1) and connected to the fingertip via an additional wire. From the outputs of the hall sensors and the number of windings around the wire pulleys, the *x* and *y* positions of the fingertip were calculated (see Section 2.4.5). The finger position was calibrated on startup by moving the finger to the upper left corner of the rectangular workspace, indicating the origin of the coordinate system.

#### 2.4.5 Software and control

All electronic components were controlled via two Teensy microcontrollers (Teensy 4.0/4.1, PJRC, United States) and connected to a personal computer via serial connections. Two separate microcontrollers were employed due to limitations in the serial communication bandwidth. One Teensy microcontroller (Teensy A) controlled the motors and brake of the tactile and kinesthetic feedback mechanisms, respectively. The other microcontroller (Teensy B) read the analog data from the hall sensors and computed the fingertip position. The personal computer ran the software managing the experimental protocol. Both microcontrollers were programmed using the Arduino IDE 2.2.1 and the experimental software in the personal computer using Processing 4.0.1.

Teensy A engaged the break and thus activated the kinesthetic feedback via a Darlington Transistor Array (ULN 2003A, STMicroelectronics, Switzerland) if the fingertip was in contact with the shape. Additionally, during the combined kinesthetic and tactile feedback condition, the tactile feedback mechanism was engaged by tensioning the wires to bring the platform in contact with the fingertip. The tangential angle between the fingertip and the shape was scaled linearly to be within the platform’s angle range, specifically between −30 and 30°. The nominal tension of the wires, determining the pressure of the platform on the fingerpad, was set experimentally in a pilot study to be well-noticeable and comfortable. The motor positions were related to the platform angle and pressure and were controlled by an open-loop proportional controller using the motor encoder outputs. Additionally, the encoder positions were sent to the personal computer at a frequency of 59 Hz. The motors were driven by a motor driver (L298N, STMicroelectronics, Switzerland) and controlled at 59 kHz.

Teensy B calculated the fingertip positions from the raw sensor output $\left({\bar{p}}_{i,\;sensor}\in \left[0,1024\right];i=x,y\right)$ and the number of windings around the pulley (*n*_*i*,*pulley*_), yielding the fingertip positions as $p_{i,\;sensor}={\bar{p}}_{i,\;sensor}+n_{i,pulley}\cdot 1024-d_{i}$ , where *d*_*i*_ is the initial offset of the sensors at the origin of the experimental area. The fingertip positions were sent to the personal computer at a frequency of 59 Hz.

The experimental software on the personal computer generated the shapes and used the fingertip position data to establish whether the fingertip was in contact with the shape and at what tangential angle w.r.t. the shape. The received fingertip positions from Teensy B are transformed to match the size of the experimental area using the *map* function, with coordinates ranging between [0, 600] px in *x* and [0, 400] px in *y*. Following, the shape-fingertip tangential contact angle and whether the fingertip was in contact with the shape was sent to Teensy A at a frequency of 12 Hz, a relatively low rate due to the bandwidth of the serial connection. The experimental software was additionally used to calibrate the fingertip position, select the experimental condition, select the experiment phase—i.e., familiarization, exploration, and reproduction—and log the data at a frequency of 59 Hz.

### 2.5 Outcome metrics

#### 2.5.1 Shape exploration behaviour

To evaluate the effect of the different haptic feedback types on participants’ exploration behavior, we recorded the time participants spent exploring each shape before reproducing them (Exploration Time).

#### 2.5.2 Shape reproduction performance

To evaluate how accurately participants could reproduce each shape after the exploration phase, we measured the spatial error between the rendered and reproduced shapes. Note that participants were asked to explore and reproduce each shape three times, where one exploration with the following reproduction is referred to as a trial. Since the participants were asked to reproduce the shapes as accurately as possible but not correctly locate them in space, we subtracted the means of the shape and reproduction trajectory in the *y*-direction for their comparison. Additionally, since participants reproduced the shape at different speeds, dynamic time warping (DTW) was employed to measure the Euclidean distance between the measured and rendered shapes (Reproduction Error). We also recorded the time it took to reproduce the shapes during each of the three reproduction trials (Reproduction Time).

#### 2.5.3 Questionnaires

We compared the impact of the two types of haptic feedback on the users’ affect by evaluating their workload and motivation for each condition. We suspected that adding tactile feedback on top of kinesthetic feedback would lead to participants requiring less effort and feeling more motivated while exploring and reproducing the shapes compared to kinesthetic feedback alone. To test this hypothesis, we measured the participants’ workload using the RTLX and the motivation with a subset of statements from the IMI. In particular, we selected a subset of statements containing the subcategories for perceived competence, effort/importance, and interest/enjoyment. These three subcategories of the IMI were chosen as they were easily relatable to the performed activity and provided a broad overview over multiple aspects of the participants’ motivation. The RTLX and IMI scores were scaled from 1–21 and 1–7 to 0–100, respectively. Low values for the scaled RTLX relate to low demand and good performance, while high values for the scaled IMI relate to high perceived competence, importance, and enjoyment.

### 2.6 Statistical analysis

We recorded a total of 384 reproduction trials (sixteen participants x four shapes x three repetitions x two conditions). One trial consisted of an exploration attempt where we calculated the measured exploration time and a shape reproduction with measured reproduction time and error. For each outcome metric, we excluded outliers per condition and shape, i.e., values <Q1−1.5IQR or values >Q3+1.5IQR , where *IQR* is the interquartile range, and *Q1* and *Q3* are the first and third quartiles, respectively. The validity of the outlier removal method was verified via histogram analysis.

Since participants had unlimited time to explore the shapes before each reproduction trial, the accuracy of the reproduction of the shape could be related to the duration of the exploration. To evaluate if such a relationship indeed exists, we evaluated the correlation between the exploration time and the reproduction error and time using Spearman’s rank correlation tests.

We employed linear mixed models (LMEs) using the *lmerTest* package (Kuznetsova et al., 2017) in R to evaluate whether participants performed differently during shape exploration or shape reproduction depending on the haptic feedback condition they were allocated to, i.e., kinesthetic feedback (K) or kinesthetic + tactile (KT), the shape they were exploring and reproducing (Shapes 2–5), and the trial repetition (1–3). We chose LMEs since they are robust to violations of model assumptions, such as data that are not normally distributed (Schielzeth et al., 2020). We included the shape as a main effect to investigate whether the participants’ performance differed between shapes since some shapes may have been easier to explore and reproduce regardless of the feedback condition. The trial repetition was added as a main effect to investigate whether learning effects existed between the three repetitions for each shape. Furthermore, we investigated the interaction effect between shapes and feedback conditions to establish whether some shapes are easier to explore and reproduce depending on the provided feedback. To account for inter-participant variability, we incorporated participants as a random effect.

Post hoc multiple comparison analyses were performed when significant effects were found using the *emmeans* package with FDR correction (Benjamini and Yekutieli, 2001) for multiple comparisons. The Q-Q plots of the model residuals vs. the normal distribution were visually inspected for each performance variable to evaluate model assumptions for normality and equal variance. Further, we calculated the Shapiro-Wilk normality test (Shapiro and Wilk, 1965) for the residuals of the LME for each performance variable.

To establish whether there were differences between the haptic feedback conditions in the participants’ perceived workload and motivation, the participants’ RTLX and IMI scores were tested using the one-sided Wilcoxon signed-rank test. Here, the mean total RTLX and IMI scores and their respective subcategories were evaluated.

All statistical tests were performed in *R* (R Studio, version 2022.07.1). The significance level was set to *α* = 0.05. For significant results, the effect size was reported as the *η* ^2^ of the LMEs and as Cohen’s D for the *post hoc* analysis.

## 3 Results

Out of 384 measurements per outcome measure, the outlier removal led to 37 measurements removed for the exploration time, 20 for the reproduction error, and 33 for the reproduction time. The removed outliers did not represent excellent reproduction performances; they consisted predominantly of large exploration and reproduction times (larger than 30 s and 60 s, respectively) and extreme reproduction errors. The mean and confidence intervals of the reproductions per condition (K and KT) for Shapes 2–4 are depicted in Figure 2.

We found a significant but weak positive monotonic correlation between exploration and reproduction times (*ρ* = 0.16, *p* = 0.0025). However, we did not find a significant correlation between exploration time and reproduction error (*p* = 0.12).

### 3.1 Exploration time

**FIGURE 4:**
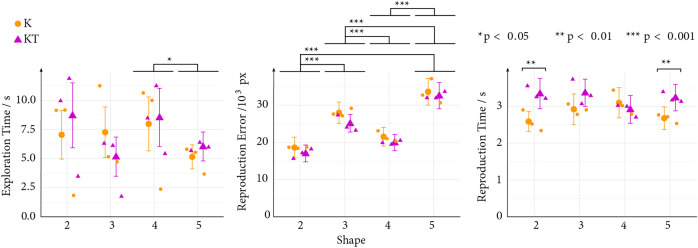
Experiment results displaying the means (markers) and 95% confidence intervals (whiskers) of the exploration time, reproduction error, and reproduction time for each shape. The small markers represent the mean of each repetition trial per condition, with repetition trials 1–3 displayed from left to right.

We found a significant effect of shape (*F*_(3,322)_ = 3.42, *p* = 0.018, *η* ^2^ = 0.03) and trial repetition (*F*_(2,322)_ = 19.34, *p* < 0.0001, *η* ^2^ = 0.11) on the exploration time (Figure 4) while the main effect of condition (i.e., Kinesthetic [K] vs. Kinesthetic + Tactile [KT] feedback) and the interaction effect between condition and shape were not significant (*F*_(1,322)_ = 0.16, *p* = 0.69 and *F*_(3,322)_ = 1.57, *p* = 0.20, respectively). The *post hoc* analysis of the shapes revealed a significant difference in exploration time between Shape 4 and Shape 5 (*p* = 0.018, *D* = 0.45), while no significant difference between the other shapes was found. Finally, the *post hoc* analysis of the trial repetition revealed that participants spent significantly more time exploring during the first and second repetition than in the third repetition (*p* < 0.0001, *D*_*t*1−*t*3_ = 0.77, *D*_*t*2−*t*3_ = 0.74). However, the interpretation of the results should be treated with caution as the normality assumption was violated (Shapiro-Wilk test, *p* < 0.001).

### 3.2 Reproduction error

We found a small but significant main effect of feedback condition on the reproduction error (*F*_(1,339)_ = 4.12, *p* = 0.044, *η* ^2^ = 0.01; Figure 4). Participants reproduced the shapes more accurately after exploring the shapes with kinesthetic and tactile feedback compared to kinesthetic feedback alone. We also found a significant effect of the shape (*F*_(3,339)_ = 53.62, *p* < 0.0001, *η* ^2^ = 0.32). However, no significant main effect on the trial repetition (*F*_(2,339)_ = 0.0044, *p* = 1.00) and interaction effects between conditions and shapes (*F*_(3,339)_ = 0.12, *p* = 0.95) were found. *Post hoc* analysis of the shape main effect showed a significant difference between all shapes (*p* < 0.0001, *D*_*S*2−*S*3_ = −1.00, *D*_*S*2−*S*5_ = −1.73, *D*_*S*3−*S*4_ = 0.68, *D*_*S*3−*S*5_ = −0.72, *D*_*S*4−*S*5_ = −1.40), except between Shape 2 and Shape 4 (*p* = 0.11). The Q-Q plot of the model residuals vs. the normal distribution indicates a normal distribution for the reproduction error, contrary to the results of the Shapiro-Wilk test, indicating a non-normal distribution (*p* < 0.001).

### 3.3 Reproduction time

We found a significant main effect of the condition and trial repetition on the reproduction time (*F*_(1,326)_ = 9.62, *p* = 0.0021, *η* ^2^ = 0.03 and *F*_(2,326)_ = 4.35, *p* = 0.013, *η* ^2^ = 0.03, respectively; Figure 4). Participants spent more time reproducing the shapes after exploring with kinesthetic + tactile feedback than exploring only with kinesthetic feedback. We did not find a significant effect on the shapes (*F*_(3,326)_ = 0.66, *p* = 0.58). However, we found a significant interaction between condition and shape (*F*_(3,326)_ = 3.27, *p* = 0.021). In particular, the *post hoc* analysis revealed that participants spent more time reproducing Shapes 2 and 5 after exploring with kinesthetic + tactile feedback than only with kinesthetic feedback (*p* = 0.0015, *D* = −0.68 and *p* = 0.0030, *D* = −0.47, respectively). Further, compared to the first trial repetition, participants spent less time reproducing the shapes in the second and third trial repetitions (*p* = 0.041, *D* = 0.32 and *p* = 0.023, *D* = 0.35, respectively). The Q-Q plot of the model residuals vs. the normal distribution indicates a normal distribution for the reproduction time, contrary to the results of the Shapiro-Wilk test, indicating a non-normal distribution (*p* < 0.001).

### 3.4 Questionnaires

We did not find significant differences between exploration conditions for the total and subcategories of the RTLX, nor for the IMI and the three evaluated subcategories (perceived competence, effort/importance, and interest/enjoyment). The resulting median scores, first and third quartiles (*Q1* and *Q3*, respectively) and *p*-values for both questionnaires and both exploration conditions (K and KT) are summarized in Table 1.

**TABLE 1:** Data analysis for the RTLX and IMI questionnaires.

	Condition K median (Q1–Q3)	Condition KT median (Q1–Q3)	K–KT *p*-value
**RTLX Total**	**41.67 (34.72–47.22)**	**41.27 (37.3–46.43)**	0.72
Mental Demand	45.24 (32.14–63.1)	42.68 (32.14–58.33)	0.18
Physical Demand	47.62 (27.38–57.14)	52.38 (38.1–58.33)	0.55
Temporal Demand	19.05 (4.76–40.48)	16.67 (4.76–29.76)	0.21
Performance	50.0 (36.9–64.29)	54.76 (41.67–58.33)	0.81
Effort	64.29 (52.38–72.62)	61.9 (51.19–72.62)	0.58
Frustration	23.81 (14.28–39.29)	28.57 (11.91–47.62)	0.89
**IMI Total**	**72.22 (57.74–75.99)**	**73.41 (61.9–80.16)**	0.30
Interest & Enjoyment	75.0 (55.36–85.71)	75.0 (55.36–85.71)	0.92
Perceived Competence	54.76 (47.62–67.86)	64.29 (41.67–67.86)	0.61
Effort & Importance	76.19 (57.14–95.24)	78.57 (70.24–91.67)	0.33

The total questionnaire scores (RTLX and IMI) are in bold, and the respective questionnaire subsection scores are below them.

## 4 Discussion

We evaluated the advantages of multimodal tactile and kinesthetic feedback on shape perception compared to kinesthetic feedback alone by conducting a pilot experiment with sixteen participants who were asked to reproduce virtual shapes after exploring them without visual feedback and with either congruent tactile and kinesthetic feedback or with only kinesthetic feedback. To facilitate this experiment, we developed a fingertip mechanism capable of providing congruent tactile and kinesthetic feedback to enhance the perception of virtual shapes. Following, we discuss the most important findings.

### 4.1 The combination of tactile feedback during virtual shape exploration together with kinesthetic feedback enhances the accuracy in reproducing virtual shapes

As hypothesized, we found that exploring virtual shapes with additional tactile feedback provided by the custom platform at the fingertip reduces reproduction errors, potentially due to participants better perceiving the shapes rendered by our device since more sensory feedback was provided (Sigrist et al., 2013). Yet, the effects of the addition of the tactile platform are smaller than anticipated. This aligns with previous studies that found improved surface perception with multimodal haptic feedback (Frisoli et al., 2011) compared to single kinesthetic or tactile feedback. Additionally, evidence of tactile feedback enabling surface curvature discrimination supports our results (Dostmohamed and Hayward, 2005). However, contrary to our expectations, we did not find shorter exploration times with the addition of tactile feedback. This suggests that training with our multisensory feedback does not seem to reduce the time participants need to feel confident enough to reproduce the explored shape.

We also found significantly higher reproduction times, although with small effects, for the condition with tactile and kinesthetic feedback compared to the kinesthetic-only feedback condition. We argue that higher reproduction times might correspond to participants being probably more careful in reproducing the shapes. We also found that participants in the multimodal haptic feedback condition spent significantly more time reproducing certain shapes compared to participants in the kinesthetic-only condition; in particular, the shape with only subtle curvatures and amplitude (Shape 2) and that with the largest amplitude and greatest reproduction errors (Shape 5). We argue that these shapes were the most difficult to reproduce despite the different reproduction errors between them. The low reproduction error observed in Shape 2 was probably due to the small amplitudes, i.e., a straight line would result in a low reproduction error anyway, while the high reproduction error in Shape 5 might be explained by the highest amplitudes. The higher reproduction times for the two most difficult shapes support our idea that participants in the multimodal haptic feedback condition were particularly careful in reproducing the shapes, especially those notably more challenging.

Finally, the significantly lower exploration and reproduction times between trial repetitions show that participants explored and reproduced the shapes faster with each repetition. However, we did not observe an improvement in the reproduction error, suggesting that participants’ reproduction performance plateaued after the first trial. This could be related to the unlimited exploration time provided to participants, where participants were free to spend as much time as they felt necessary to perceive the shapes. Since we did not find a correlation between the time spent exploring the shapes and the reproduction error, individual differences in exploration time did not contribute to better reproduction performance, indicating ceiling effects in reproduction performance, which may be due to limitations in the rendering fidelity of the respective haptic mechanisms. This is supported by the weak positive correlation between exploration and reproduction time, suggesting that a participant’s rigor in exploring can also be seen in the reproduction, i.e., participants who were more careful exploring were also more careful reproducing—without better reproduction performance.

### 4.2 Exploring shapes with the addition of tactile feedback did not increase motivation nor decrease workload

Since the additional tactile feedback probably provided a richer experience than kinesthetic feedback alone, we hypothesized that the multimodal haptic feedback would lower the users’ workload and increase their motivation. However, contrary to our hypothesis, we found no significant difference in these affects between exploration conditions.

Looking at the absolute workload, the values between the first and third quartile (*Q1*-*Q3*) are within or below the band from 25–75, indicating an intermediate workload level (Ranzani et al., 2021). Furthermore, the perceived temporal demand and frustration partially indicated minimal workload (median and *Q1* < 25). Therefore, the lack of differences between conditions found in the workload questionnaire may be due to the exploration task being perceived as relatively low demanding with the kinesthetic feedback only, limiting potential benefits resulting from the additional provision of tactile feedback. The beneficial effects of multisensory feedback on performance may become apparent in more demanding tasks.

When analyzing the IMI responses, we observed high scores (median of 72 in the kinesthetic-only condition and 73 in the multimodal haptic condition), especially in the subscales of interest/enjoyment and effort/importance. In comparison, the reported values in perceived competence were lower (median of 55 in the kinesthetic-only condition and 64 in the multimodal haptic condition). While the reported perceived competence was 10 points higher in the multimodal haptic feedback condition compared to participants in the kinesthetic-only condition—in line with their better reproduction performance—the difference was not significant, probably due to the small sample size and high variability in the dataset.

It is important to note that we did not provide any type of terminal feedback regarding the participants’ performance during the shape reproduction. The addition of terminal feedback might affect participants’ cognitive load and subjective motivation, especially the perceived competence. It is an open question if the addition of terminal feedback may increase participants’ cognitive load and affect their motivation, making the differences between conditions more salient.

### 4.3 Study limitations and future work

There are several limitations regarding the conducted experiment. The first limitation is the small number of participants included in this pilot study. A larger number of participants should be included to increase the statistical power of the experiment. Additionally, while we report the participants’ inexperience with the haptic device used in this study, information on their previous experience with haptic interfaces should be obtained, as this could influence their performance.

Second, we investigated the usefulness of kinesthetic feedback and the addition of tactile feedback, but not for tactile feedback alone. This prohibits us from making any statements on the usefulness of tactile feedback alone for shape exploration and reproduction. We did not include a tactile feedback only condition for two reasons. First, our goal was to improve the performance of current commercial kinesthetic devices, like the SenseGlove Nova used in our study, to see if these enhancements could better shape perception and, eventually, enhance haptic feedback in VR training. Second, we included 1D curves in our study with curvature and amplitude information. We anticipated that with only tactile feedback, participants would be unaware of the shapes’ amplitude and location, limiting the comparison’s value.

Further, we did not constrain the participants within the two-dimensional rectangular area. Although participants were instructed to rest their palms on the sliding guide and try not to lose contact with it, they still could move their hands if desired. This could have led to potential shifts or transformations of the rendered virtual shape. Additionally, we calculated an approximated fingertip position as a linear function of the individual wire lengths of the position sensors, one for *x* and one for *y*; however, this is inaccurate since the fingertip position depended on both wire lengths. Regardless, since the rendered and recorded shapes were based on the same approximated fingertip positions, the effect of this approximation only marginally influenced our results.

Another limitation is the tendon-based transmission of the tactile feedback mechanism. As the wires from the motors directly interacted with the guides on the dorsal side of the finger and the platform, they exerted a residual force on the entire finger instead of just actuating the platform. Estimating the forces that acted on the finger, assuming that the springs for the platform were halfway compressed and the cables had an inclination of 15°, yields vertical forces below 1 N. These forces may be negligible compared to the break forces of the kinesthetic feedback. In hindsight, this issue could have been avoided using sheathed cables like Bowden cables.

Moreover, the communication frequency between the experimental software and the microcontroller controlling the platform angle and brake engagement was limited due to the serial bandwidth. Regardless, while testing, we did not notice any issues from the limited update rate, likely due to the slow finger movements of the users, which are usually under 10 Hz (Antonsson and Mann, 1985).

Finally, while we collected information regarding workload and motivation through questionnaires, we did not collect feedback regarding the system usability, how participants perceived the two feedback modalities, and whether they perceived those congruently. This kind of feedback could be valuable for future device developments.

Future work should focus on a refined experimental setup, particularly the force transmission of the tactile interface and the fingertip tracking. This refined experimental setup should be developed following a human-centered design approach, including formal usability assessments. Furthermore, alternative tactile stimuli, such as vibrations or normal forces, should be explored and compared to the presented solution regarding shape perception.

## 5 Conclusion

In this study, we investigated virtual shape perception and reproduction under two haptic conditions—kinesthetic feedback and combined kinesthetic and tactile feedback as a means to enhance virtual reality training environments. We found that providing tactile and kinesthetic feedback during a virtual shape exploration task without visual feedback is associated with more accurate and careful shape reproduction compared to exploring shapes with only kinesthetic feedback. However, the addition of tactile feedback does not seem to reduce the time spent during exploration, nor does it have an effect on motivation or workload. Thus, combining tactile and kinesthetic feedback could create more realistic virtual environments, possibly leading to better training results and easier transfer to real-world tasks, which merits further study.

## Data Availability

The datasets presented in this study can be found in online repositories. The names of the repository/repositories and accession number(s) can be found below: The data is available in the open repository Zenodo (Ratschat et al., 2024).
